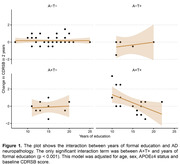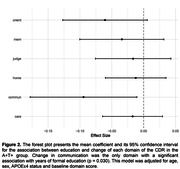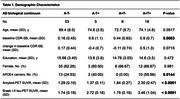# Interaction between Education and Alzheimer’s Disease Biomarkers in Longitudinal Clinical Impairment

**DOI:** 10.1002/alz.093545

**Published:** 2025-01-09

**Authors:** Thomas Hugentobler Schlickmann, Wyllians Vendramini Borelli, João Pedro Uglione da Ros, Luiza Santos Machado, Firoza Z Lussier, Arthur C. Macedo, Nesrine Rahmouni, Joseph Therriault, Stijn Servaes, Jenna Stevenson, Mira Chamoun, Gleb Bezgin, Andrea L. Benedet, Pedro Rosa‐Neto, Eduardo R. Zimmer

**Affiliations:** ^1^ Universidade Federal do Rio Grande do Sul, Porto Alegre, Rio Grande do Sul Brazil; ^2^ Memory Center, Hospital Moinhos de Vento, Porto Alegre, RS Brazil; ^3^ Lutheran University of Brazil, Canoas, Rio Grande do Sul Brazil; ^4^ University of Pittsburgh, Pittsburgh, PA USA; ^5^ Translational Neuroimaging Laboratory, The McGill University Research Centre for Studies in Aging, Montréal, QC Canada; ^6^ University of Gothenburg, Gothenburg Sweden

## Abstract

**Background:**

Higher education is often associated with reduced risk of cognitive decline. These findings fueled the conceptualization of cognitive reserve to explain individual variabilities in clinical trajectory. However, our understanding of the biological basis for this phenomenon is still not precise. In this sense, we assessed the interaction between Alzheimer’s biomarkers and cognitive reserve.

**Method:**

We included 85 individuals from the Translational Biomarkers of Aging and Dementia (TRIAD) cohort presenting normal cognition or mild cognitive impairment with global Clinical Dementia Rating (CDR) ≤ 0.5. All individuals underwent positron emission tomography (PET) for amyloid‐β (A) and tau (T) assessed with [18F]AZD4694‐PET and [18F]MK6240‐PET, respectively. A positivity was defined as neocortical Aβ‐PET SUVR ≥ 1.55. T positivity was defined as temporal meta‐ROI tau‐PET SUVR ≥ 1.24. We calculated a change in CDR sum of boxes (CDR‐SB) by subtracting baseline from their scores after 2 years of follow‐up. Analysis were conducted with the CDR sum of boxes (CDRSB) as well as within specific domains, always correcting for age, sex, *APOEε4* carriership and baseline score.

**Result:**

Demographics information is described in **Table 1**. Linear regression analysis showed that years of education interacted with A+T+ status to reduce cognitive decline (β = ‐0.19, p < 0.001) but did not interact with any other pathological status as shown in **Figure 1**. We also found that communication, a specific domain of the CDR scale, had a longitudinal association with education in the A+T+ group (β = ‐0.09, p = 0.0303, **Figure 2**).

**Conclusion:**

The protective effect of education, as measure of cognitive reserve, occurred specifically in A+T+ individuals. Moreover, this effect was mostly on the communication domain. These results contribute to our understanding of cognitive reserve as they may suggest that education protects communication from the synergistic damage of amyloid and tau pathologies. Further investigations in additional cohorts with longer follow‐up is warranted.